# Designing High‐Sensitivity Mechanochromic Luminescent Materials Through Friction‐Induced Crystallization Strategy

**DOI:** 10.1002/advs.202409974

**Published:** 2024-10-21

**Authors:** Zhihang An, Zhenhao Dai, Jiaping Liu, Si Chen, Xu Wang, Heyang Liu, Zhongyi Sheng, Tianyu Shan

**Affiliations:** ^1^ College of Biological & Chemical Engineering Zhejiang University of Science and Technology Hangzhou 310023 P. R. China; ^2^ Stoddart Institute of Molecular Science, Department of Chemistry Zhejiang University Hangzhou 310058 P. R. China; ^3^ College of Environmental and Natural Resources Zhejiang University of Science and Technology Hangzhou 310023 P. R. China; ^4^ College of Materials Science and Engineering Zhejiang University of Technology Hangzhou 310014 P. R. China

**Keywords:** aggregation‐induced emission, friction‐induced crystallization, information encryption, mechanochromic luminescence, stimuli response

## Abstract

Despite recent significant breakthroughs in novel mechanochromic luminescent (ML) materials, developing a high‐sensitivity ML material is still challengeable. Herein, a “friction‐induced crystallization” strategy is proposed to realize highly sensitive transformations of luminescent signal, through an integration of polymeric chains and an aggregation‐sensitive luminescent core, which act as mechanical sensors and fluochromic actuators, respectively. The coupling of these two components enables the material to crystallize in response to shear friction, thereby exhibiting blue‐shift fluorescence due to a more restricted relaxation pathway. This study underscores a high‐sensitivity ML material based on the precise regulation of molecular‐scale motions, and also expands the scope and potential of ML materials toward user‐friendly, interactive wearable devices.

## Graphical abstract

The authors propose a “friction‐induced crystallization” strategy to realize the high‐sensitivity mechanochromic luminescent material by applying shear friction, through an integration of polymeric chains and an aggregation‐sensitive luminescent core. This research extends the scope and potential of mechanochromic luminescent materials, and shows a multilevel information encryption on flexible fabrics.

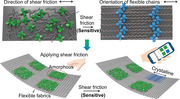

## 1 Introduction

Mechanochromic luminescent (ML) materials,^[^ ^1^, ^2^ ^]^ which exhibit emission changes in response to external mechanical stimuli, have garnered significant attention due to their scenarios in intelligent sensors,^[^ ^3^, ^4^, ^5^ ^]^ information encryption,^[^ ^6^, ^7^, ^8^, ^9^ ^]^ optoelectronic devices,^[^ ^10^, ^11^ ^]^ etc. Generally, mechanical impact leverages the disruption of specific intermolecular interactions to transform molecular packing mode, ultimately leading to emission changes.^[^ ^12^, ^13^ ^]^ As reported, ML materials typically involve a transformation from tight to incompact molecular packing under a certain strength of mechanical force such as grinding and scratching, corresponding to a transition from crystalline to amorphous states.^[^ ^14^, ^15^ ^]^ However, such mechanical force reflects the low‐sensitivity and restricted application scenarios of ML materials.^[^ ^16^, ^17^, ^18^ ^]^ For example, the material must be subjected to a hard substrate to disrupt its crystalline structure. Regarding to flexible substrates, the major mechanical energy will be absorbed by the substrate deformation, thereby maintaining its original molecule packing.^[^ ^19^, ^20^ ^]^ The possibility of high‐sensitivity ML material has been noted on several occasions,^[^ ^21^, ^22^ ^]^ however, the realization of this desirable material remains a challenge.

In polymer science, when subjected to shear stress, polymer chains will be regularly aligned along the direction of the stress with maximum packing density, thereby inducing crystallization.^[^ ^23^, ^24^, ^25^, ^26^ ^]^ The phenomenon is instructive as the crystallization normally implies the fluorescent change. More importantly, the sources of shear stress are variable, originating from either intense grinding or mild friction.^[^ ^27^, ^28^, ^29^ ^]^ Consequently, we hypothesize that a molecule combing polymeric chains and a rigid luminescent chromophore may show high‐sensitivity ML property, through shear friction.^[^ ^30^, ^31^ ^]^ In this process, the polymeric chains act as sensing subsystems responding to mechanical stimuli, while environment‐sensitive chromophores serve as chromic subsystems to execute luminescent change upon crystallization.^[^ ^32^, ^33^, ^34^, ^35^ ^]^ Overall, ML on flexible substrates is viable by integrating polymeric chains into luminescent molecules.

**Figure 1:**
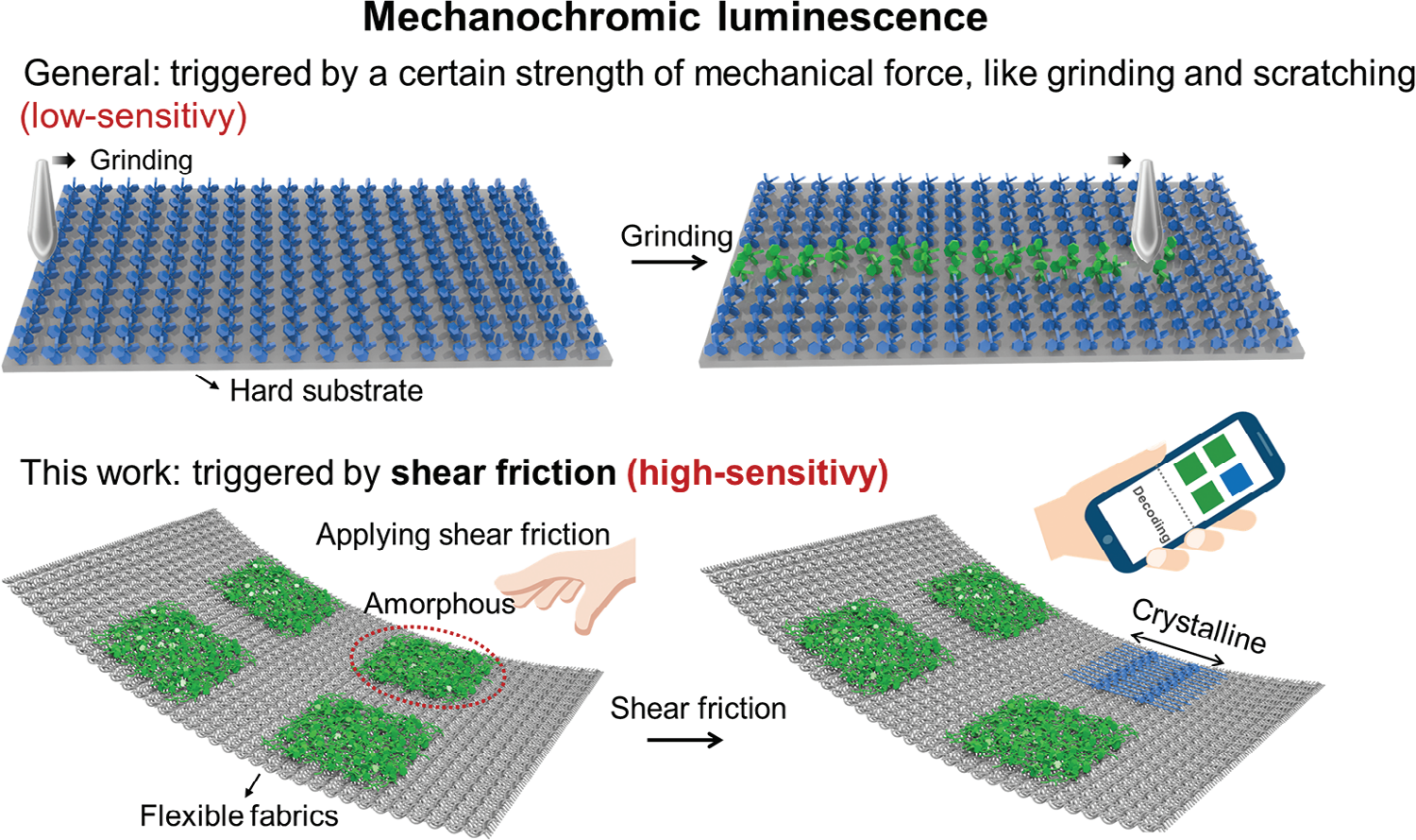
Comparison of mechanochromic luminescence with low and high sensitivity. Generally, ML is triggered by a certain strength of mechanical force to destroy the crystalline structure on a hard substrate, corresponding to a transition from crystalline to amorphous states. In this work, ML was triggered by mild friction, through the transformation from amorphous to crystalline state. Friction is high sensitivity and supports flexible scenarios. Based on these, a multilevel anti‐counterfeiting system was designed, by a customized application recognizing the fluorescent patterns.

Herein, we reported a high‐sensitivity mechanochromic luminescent material, actualized by the friction‐induced crystallization strategy. In particular, an aggregation‐induced emission generator (AIEgen), **TPE‐C12** was synthesized by connecting two dodecyloxy chains on tetraphenylethene (TPE, Figures S1–S4, Supporting Information), which integrated multiple advantages. On the one hand, the polymeric dodecyloxy chains would be regularly orientated in response to friction, leading to a tight molecule packing facilitating crystallization.^[^ ^36^, ^37^, ^38^, ^39^, ^40^ ^]^ On the one hand, as typical AIE‐active components, TPE unit exhibited aggregation‐sensitive emission, and its fluorescence is highly correlated with its molecular state.^[^ ^41^, ^42^, ^43^, ^44^, ^45^ ^]^ As a result, the combination of these two components allowed **TPE‐C12** to display high‐sensitive ML by mild friction. Different from the low‐sensitivity ML materials that requires a certain strength of mechanical force to destroy crystal structure on hard substrates, this high‐sensitivity ML material open a new door for applications on flexible substrates. As a demonstration, **TPE‐C12** was further employed for preparing encryption codes on flexible fabrics (**Figure** **1**), and the encryption system was decoded by combining heating and friction. Overall, this research highlights a high‐sensitivity ML material, extending the scope and potential of ML materials, and also provided a user‐friendly, interactive, multilevel information encryption system, contributing to next‐generation wearable devices.

## 2 Results and Discussion

**Figure 2:**
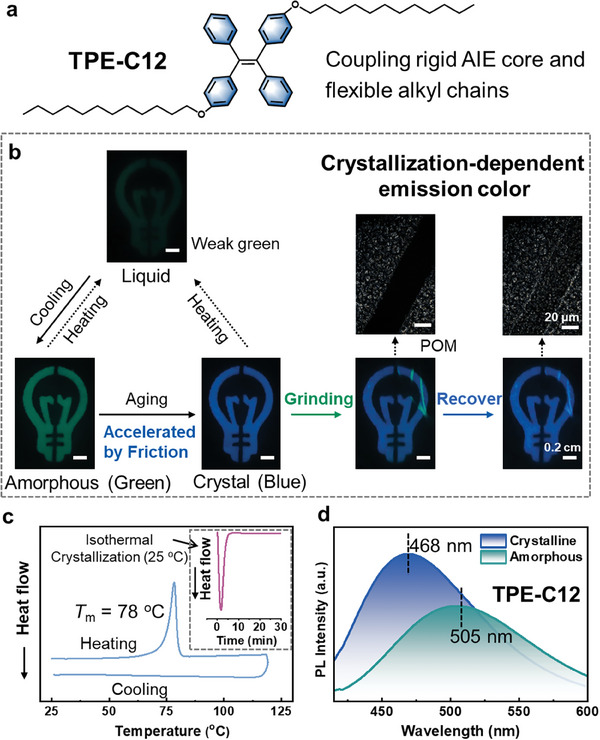
Aggregation‐sensitive emission of TPE‐C12. a) Chemical structure of **TPE‐C12**, coupling flexible alkyl chains and a rigid TPE core. b) Fluorescent characteristic and related transformations of **TPE‐C12**. At disorder state, including liquid and amorphous state, **TPE‐C12** presents green emission. At crystalline state, **TPE‐C12** emits blue color. c) Differential scanning calorimetry measurement of **TPE‐C12**, verifying the hysteretic crystallization kinetics. Inset: isothermal procedure at 25 °C. d) Fluorescence spectra of **TPE‐C12** during amorphous and crystalline state.

Originally, the luminescent properties of **TPE‐C12** have been thoroughly studied. As depicted in **Figure** **2**a, the AIEgen **TPE‐C12** coupled rigid TPE core and flexible alkyl chains, resulting in unique luminescent properties.^[^ ^46^, ^47^ ^]^ Generally, crystalline **TPE‐C12** presented blue‐emission and sharp X‐ray diffraction (XRD) peaks (Figure 2b; Figure S5, Supporting Information). After heating at 80 °C, the crystalline **TPE‐C12** melted and exhibited weak green luminescence and weak‐intensity XRD patterns (Figure S5, Supporting Information). Subsequently, the photoluminescence (PL) intensity strengthened and the green luminescence gradually converts to blue during cooling, undergoing a liquid‐amorphous‐crystalline transformation. Moreover, the crystallization‐induced blue‐shift emission was elaborately investigated. As shown in Figure 2b, grinding the **TPE‐C12** crystals converted the emission color from blue to green, corresponding to a transformation from crystal to amorphism, as revealed by polarizing optical microscope (POM). The green emission was metastable, and after a certain period of delay, the fluorescence color recovered to blue, driven by the higher degree of disorder and a more crowded space effect in amorphous state (Movie S1, Supporting Information). Meanwhile, the birefringence phenomenon was observed by POM in 4 min (Figure S6, Supporting Information), indicating the long‐range ordering of crystalline structure was self‐recovered. The self‐recovered luminescence color and crystalline structure were confirmed by differential scanning calorimetry (DSC), whose test procedure was set as follows: first, the temperature was raised to 120 °C and then cooled down to 25 °C with a rate of 10 °C/min. Ultimately, the temperature was remained at 25 °C for 30 min. As shown in Figure 2c, **TPE‐C12** melted at 78 °C (*T*_m_) by heating, but the crystallization peak was absent during cooling procedure. In contrast, a peak corresponding to cold crystallization emerged at isothermal procedure (25 °C, Figure 2c, inset), verifying the hysteretic crystallization kinetics and the self‐recovered crystalline structure at room temperature.

More importantly, the shear stress‐induced blue‐shift emission was confirmed, by stirring (Figure S7 and Movie S2, Supporting Information) and friction (Figure 2b). Particularly by applying friction, the transition from green to blue emission (505 to 468 nm, Figure 2d) could be significantly accelerated, even after grinding (Figure S8, Supporting Information), since the intermolecular rearrangements would be promoted by chain orientation. In brief, AIEgen **TPE‐C12**, associated with a balance of molecular rigidity and flexibility, exhibits hysteretic crystallization kinetics and crystallization‐dependent emission, leading to high‐sensitivity ML. Although the above green‐shift and blue‐shift fluorescence are both mechanical force‐induced, however, the mechanisms are totally different. Grinding, as a high‐strength mechanical force, would destroy the crystalline structure of **TPE‐C12**, leading to the transformation to amorphous state and green‐shifted fluorescence. Conversely, friction exerted low‐strength shear stress to **TPE‐C12**, leading to the transformation to crystalline state and blue‐shifted fluorescence. Thereby, this work also provides convertible fluorescence color through precise control of mechanical force.

**Figure 3:**
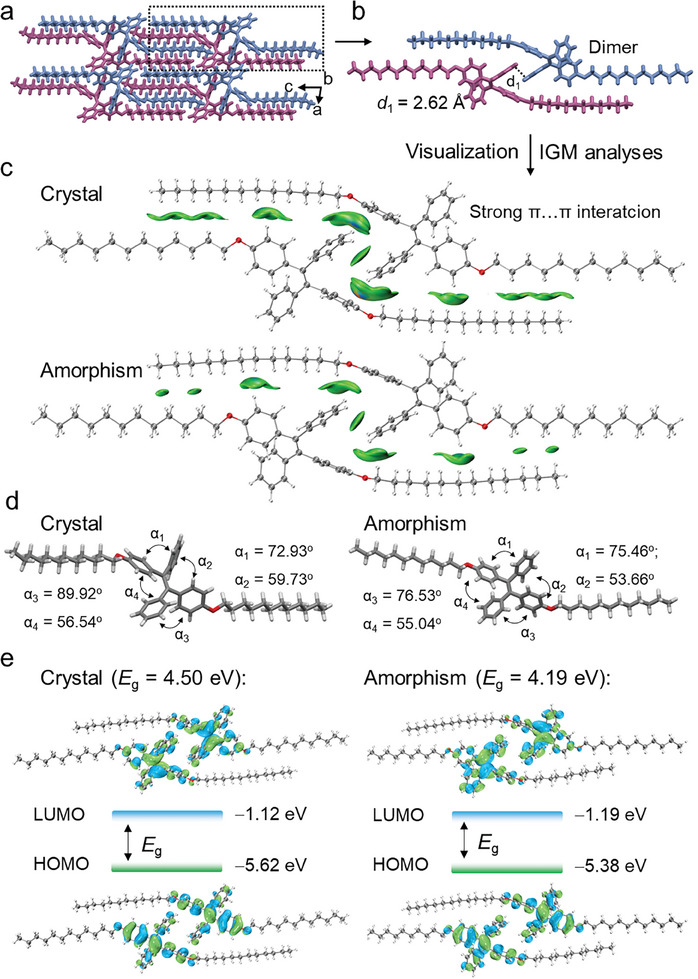
Theoretical calculations. a) Crystal structure of **TPE‐C12** at packing view, viewed along *b* axis. b) Crystal structure of **TPE‐C12** dimer. c) The independent gradient model analyses of crystalline and amorphous **TPE‐C12** dimers. Crystalline dimer present stronger intermolecular interactions. d) Crystal and amorphism structures of **TPE‐C12** molecule and related dihedral angles. Crystalline **TPE‐C12** molecule present a more twisty conformation. e) Calculations of HOMO‐LUMO gaps of crystalline and amorphous **TPE‐C12** dimers.

To gain a deeper insight into the underlying mechanism of above mechanochromic luminescent phenomenon at molecular level, theoretical calculations was subsequently performed. Initially, the single crystals of **TPE‐C12** were obtained by recrystallization in *n*‐hexane. Subsequently, single crystal X‐ray diffraction (SCXRD) was employed to obtain the precise molecular structure information of **TPE‐C12**. As displayed in **Figure** **3**a,b, **TPE‐C12** presents two conformations which further interlaced into dimers by *π–π* and aliphatic interactions. Regarding to the amorphous state, its molecular conformation was treated as gas state by applying time dependent density functional theory (DT‐DFT) calculations. The results revealed that the amorphous state showed more incompact intermolecular packing compared to crystalline one. Second, to visualize the intermolecular interactions, independent gradient model (IGM, Figure 3c) and non‐covalent interaction (NCI, Figure S9, Supporting Information) analyses of the two dimers were calculated. As shown in Figure 3c, the green patterns represented *π–π* or aliphatic interactions. Larger areas of the green patterns, i.e., stronger intermolecular interactions, were found in crystalline structure. In light of this, we speculated that the excited‐state relaxation pathway played a crucial role during the emission color transformation between amorphisms and crystals.^[^ ^48^, ^49^, ^50^ ^]^ In amorphous state, relaxation pathway was active due to weaker intermolecular interactions. However, the relaxation pathway in crystalline state was restricted by strong intermolecular interaction in lattice, resulting in a bigger gap and blue‐shift emission.^[^ ^51^, ^52^ ^]^ Besides, due to physical constraints, the conformation of the TPE core was more twisty in the crystalline state (Figure 3d; Figure S10, Supporting Information), equally facilitating blue‐emission. Finally, to verify the conjecture, the highest occupied molecular orbital‐lowest unoccupied molecular orbital (HUMO‐LUMO) energies and associated gaps (*E*_g_) were calculated and displayed in Figure 3e. The HOMO‐LUMO gaps of crystalline dimers were calculated as 4.50 ev, which is bigger than amorphous dimers, calculated 4.19 ev. Fully understanding of the emission change mechanism of **TPE‐C12** at molecular lever provides theoretical supports for designing new ML materials and their further applications.

**Figure 4:**
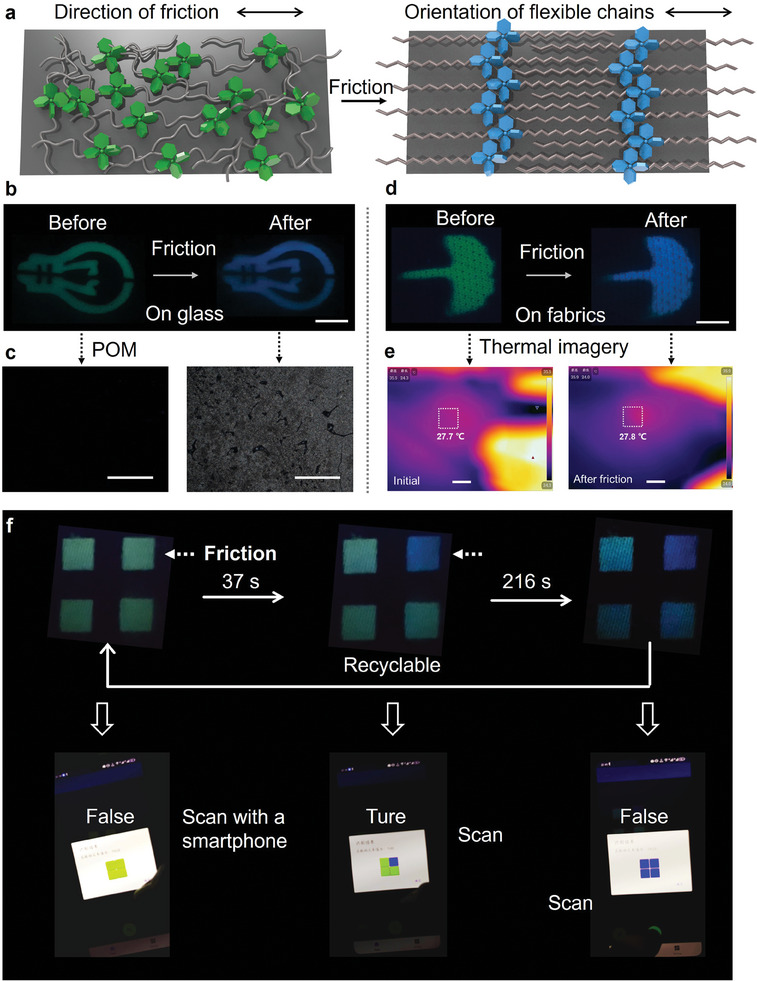
High‐sensitivity mechanochromic luminescence triggered by friction. a) Schematic diagram of friction‐accelerated crystallization and blue‐shift emission of **TPE‐C12**. Frictional shear stress induced the pre‐organization of alkyl chains, thereby accelerating the crystallization kinetics. Once amorphous **TPE‐C12** molecules crystallized, the emission color changed to blue. b) By applying shear friction, the crystallization of **TPE‐C12** was accelerated on glass. The scale bar is 10 mm. c) POM images before and after friction. The scale bars are 45 µm. d) ML of **TPE‐C12** on fabrics triggered by friction. The scale bar is 10 mm. e) Infrared thermal images captured before and after friction, revealing that the thermal effect can be neglected during friction. The scale bars are 10 mm. f) Demonstration of the multilevel information encryption by coupling heating and friction. The true information only appeared with correct decryption method.

Subsequently, the high‐sensitivity mechanochromic luminescence through friction‐induced crystallization was minutely investigated. In theory, the frictional shear stress generated at the interface, induced the pre‐organization of alkyl chains, thereby accelerating the crystallization kinetics (**Figure** **4**a). As a verification, a pattern of lamp bulb was drawn on a glass slide with the DCM solution of **TPE‐C12**, presenting green emission under 365 nm UV light immediately removing solvents by heating. By now, **TPE‐C12** adopted incompact and long‐range disordered packing, indicated by POM (Figure 4b,c). The emission color converted to blue after friction (Movie S3, Supporting Information), corresponding to a high‐density and regular packing. Then **TPE‐C12** was sprayed onto a flexible fabric, generating a green‐emission leaf pattern after heating. ML characteristic of **TPE‐C12** remained on the fabric, and the emission color converted to blue after friction as on the hard substrate (Figure 4d; Movie S3, Supporting Information). Specifically, the shear friction was applied to **TPE‐C12** by holding a fabric with the hands, and the infrared thermal imagers showed huge resistance for thermal conduction from hands to the fabric (Figure S11, Supporting Information). Meanwhile, Figure 4e shows that the temperature remains constant during the friction process, revealing that the thermal effect of friction can be neglected. These understanding of the friction‐promoted emission change offer assurance in the design and exploration of novel multilevel information encryption system based on fabrics. Due to the aggregation‐sensitivity nature of **TPE‐C12**, a multilevel encryption system could be designed combing temperature and friction (Figure S9 and Movie S4, Supporting Information). Herein, we show a model system to elucidate the fundamental principles to operate this multilevel information encryption system. As shown in Figure 4f, **TPE‐C12** was sprayed on the fabrics to form a scanning code of 2‐by‐2 squares, all emitting blue emission after crystallization. The scanning code could be recognized by a smartphone to show information by a customized application, and the true information only appeared with correct decryption method. As a model system, the decryption program was set as follows. First, heating the scanning code above 80 °C for a few seconds, such as simply using a hair dryer (Figure S12, Supporting Information). **TPE‐C12** became disordered and all the squares emitted green emission. Second, when the temperature of the fabrics dropped to around room temperature, rubbing one specific square converted its emission color to blue since the shear friction accelerates the crystallization of **TPE‐C12** (≈40 s). At this juncture, the true information was obtained by smartphone scanning (Movie S5, Supporting Information). Finally, the true information burned after reading, since the other three squares presented blue‐emission after 216 s, i.e., returning to the original state. Moreover, another scanning code of 3‐by‐3 squares with a more complex anti‐counterfeiting procedure is shown in Figure S13 and Movie S6 (Supporting Information). Remarkably, the simple decryption method, heating and friction, are implementable during daily life. One only needs a hairdryer and hands to decrypt the system, presenting great potential for real applications.

## 3 Conclusion

In summary, we report a friction‐induced crystallization strategy to design high‐sensitivity mechanochromic luminescent (ML) materials, with a luminescent molecule **TPE‐C12**, featured with a tetraphenylethene (TPE) core and two long flexible alkyl chains. The balance of molecular softness and rigidity renders **TPE‐C12** with hysteretic crystallization kinetics and crystallization‐dependent emission. More importantly, the crystallization could be significantly accelerated by applying shear friction, thereby exhibiting high‐sensitivity mechanochromic luminescence. Density functional theory (DFT) calculations revealed that the crystalline **TPE‐C12** adopts a more compact intermolecular packing stabilized by stronger intermolecular interactions, which restricted the relaxation pathway and led to a bigger gap and blue‐shift emission. Besides, the high‐sensitivity ML material was sprayed onto fabrics to prepare multilevel information encryption, decoded by facile heating and friction. We anticipate that this friction‐induced crystallization strategy proposed here can inspire the further design and engineering of the next‐generation high sensitivity mechanochromic luminescent materials for more shining applications.

## 4 Experimental Section

### Materials

**TPE‐C12** was synthesized in the laboratory according to the synthetic route in supporting information. All reagents and solvents were purchased from Aladdin (Shanghai, China) with analytical purity, and used as purchased without further purification.

### Differential Scanning Calorimeter

The samples were heated (from 25 to 120 °C) and then cooled (from 120 to 25 °C) in a nitrogen atmosphere with a scan rate of 10 °C min^−1^. Subsequently, the temperature was remained at 25 °C for 30 min to observe the isothermal crystallization.

### Theoretical Calculations

Frontier molecular orbitals were calculated using TD‐DFT method at B3LYP‐D3(BJ)/6‐31G(d,p) level, Gaussian 09 program.^[^ ^53^ ^]^ The independent gradient model (IGM), non‐covalent interaction (NCI) analyses, and reduced density gradient (RDG) analysis were performed using Multiwfn program to visualize the intermolecular interaction.^[^ ^54^ ^]^ The visualization of the mapping of IGM and RDG were all rendered using VMD program.^[^ ^55^ ^]^.

### The Preparation of Fluorescent Patterns

The solid **TPE‐C12** was weighed according to the calculated amount and solved in CH_2_Cl_2_ to obtain a solution with a concentration of 1 mg mL^−1^. Then the solution was sprayed on glass or dust‐free cloth with N_2_ blowing. The fluorescent patterns were produced by specific templates during the spray process, including light bulbs, leaves and squares. And the “smart”’ scanning codes of 2, 2‐by‐2, and 3‐by‐3 squares were produced by the same method. The size of each square is 1 × 1 cm.

### Customized Application

The customized application could be downloaded onto an Android smartphone to accurately identify the blue and green squares of the scanning codes. The coded information (the word “True”) could only be read when the blue square appears at the correct coordinates, otherwise an error message was displayed (the word “False”).

## Conflict of Interest

The authors declare no conflict of interest.

## Supporting information



Supporting Information

Supplemental Movie 1

Supplemental Movie 2

Supplemental Movie 3

Supplemental Movie 4

Supplemental Movie 5

Supplemental Movie 6

Supporting Information

## Data Availability

The data that support the findings of this study are available from the corresponding author upon reasonable request.
