# Supplementary material for: Designing High‐Sensitivity Mechanochromic Luminescent Materials Through Friction‐Induced Crystallization Strategy
Source: Adv Sci (Weinh). 2024 Oct 21;11(46):2409974. doi: 10.1002/advs.202409974 (PMC11633541; doi:10.1002/advs.202409974)
Supplement: Supplementary file 1 — Supporting Information [file ADVS-11-2409974-s004.pdf]

ADVANCED SCIENCE
Open Access

Supporting Information

for *Adv. Sci.*, DOI 10.1002/advs.202409974

Designing High-Sensitivity Mechanochromic Luminescent Materials Through Friction-Induced Crystallization Strategy

Zhihang An, Zhenhao Dai, Jiaping Liu, Si Chen, Xu Wang, Heyang Liu, Zhongyi Sheng* and Tianyu Shan**

Supporting information

Designing High-Sensitivity Mechanochromic Luminescent Materials through Friction-Induced Crystallization Strategy

Zhihang An,[1] Zhenhao Dai,[1] Jiaping Liu,[1] Si Chen,[4] Xu Wang,[4] Heyang Liu,[13]* Zhongyi Sheng,[1]* and Tianyu Shan[2]*

[1] College of Biological & Chemical Engineering, Zhejiang University of Science and Technology, Hangzhou, 310023, P. R. China

[2] Stoddart Institute of Molecular Science, Department of Chemistry, Zhejiang University, Hangzhou 310058, P. R. China

[3] College of Environmental and Natural Resources, Zhejiang University of Science and Technology, Hangzhou, 310023, P. R. China

[4] College of Materials Science and Engineering, Zhejiang University of Technology, Hangzhou 310014, P. R. China

* Corresponding authors. Email: heyang.liu@zust.edu.cn; shengzyhz@zust.edu.cn; tyshan@zju.edu.cn;

Table of Contents

1. Instrumentation and Methods S2

2. Figures S1 to S14..S4

3. Table S1 ....S10

Section 1. Instrumentation and Methods

1.1 Instrumentation

^{1}H and ^{13}C nuclear magnetic resonance (NMR) spectra were recorded on a Bruker AVANCE III (500MHz) NMR spectrometer in $CDCl_3$. Chemical shifts are reported in ppm relative to the residual nondeuterated solvent signals ($CHCl_3$: δH = 7.26 ppm and δC = 77.1 ppm). Mass spectrometry (MS) was performed on a Bruker ultrafleXtrem matrix-assisted laser desorption ionization time-of-flight (MALDI-TOF) mass spectrometry. Differential scanning calorimeter (DSC) curves were recorded on a DSC Q100 V9.7 Build 291 differential scanning calorimeter. Single crystal X-ray diffraction (SCXRD) data were obtained on an Oxford Gemini A Ultra diffractometer (Mo-Kα radiation, λ = 0.71073 Å, Atlas CCD detector). Photoluminescence spectra were obtained using a QEPro2064 optical spectrometer. Polarized optical microscope (POM) images were recorded on a CX4OP microscope. Thermal images were recorded on a FOTRIC323Pro professional handheld thermal imager.

1.2 TGA

Thermogravimetric analysis (TGA) was carried out on a SDT Q600 thermogravimetric analyzer. The samples were heated in an air atmosphere with a scan rate of 10°C/min.

1.3 Synthesis of TPE-C12

In a 50 mL round-bottomed flask, 1-bromododecane (2.73 g, 11.0 mmol), 4-[2-(4-hydroxyphenyl)-1,2-diphenylethenyl]phenol (1.00 g, 2.74 mmol) and K_2CO_3 (2.73 g, 11.0 mmol) were added in 30 mL acetone. Then the mixture was stirred and refluxed for 16 h at 80 °C. After the reaction was completed, the solution was added into 30 mL ethyl acetate and washed with 0.5 M HCl aqueous solution and deionized water separately. The organic layer was dried over anhydrous Na_2SO_4, filtered, and the volatiles were evaporated in vacuo. The crude product was purified by chromatography on silica gel with petroleum ether and dichloromethane to afford **TPE-C12** as white powders (1.44 g, 75%). Melting point: 78 ºC. ^{1}H NMR ($CDCl_3$, 500 MHz, 298K): δ

7.14–7.06 (m, 10H), 6.92 (d, 4H), 6.63 (d, 4H), 3.88 (t, 4H), 1.74 (m, 4H), 1.43 (m, 4H), 1.28 (m, 32H), 0.90 (t, 6H). ^{13}C NMR ($CDCl_3$, 500 MHz, 298K): δ 157.5, 144.3, 139.6, 136.2, 132.5, 131.4, 127.5, 126.1, 113.5, 67.8, 34.0, 32.8, 31.9, 29.6, 29.5, 29.4, 28.8, 28.2, 26.1, 22.7, 14.1. MALDI-TOF-MS: m/z calculated for $[M]^+$ $C_{50}H_{68}O_2^+$ 700.522, found 700.680.

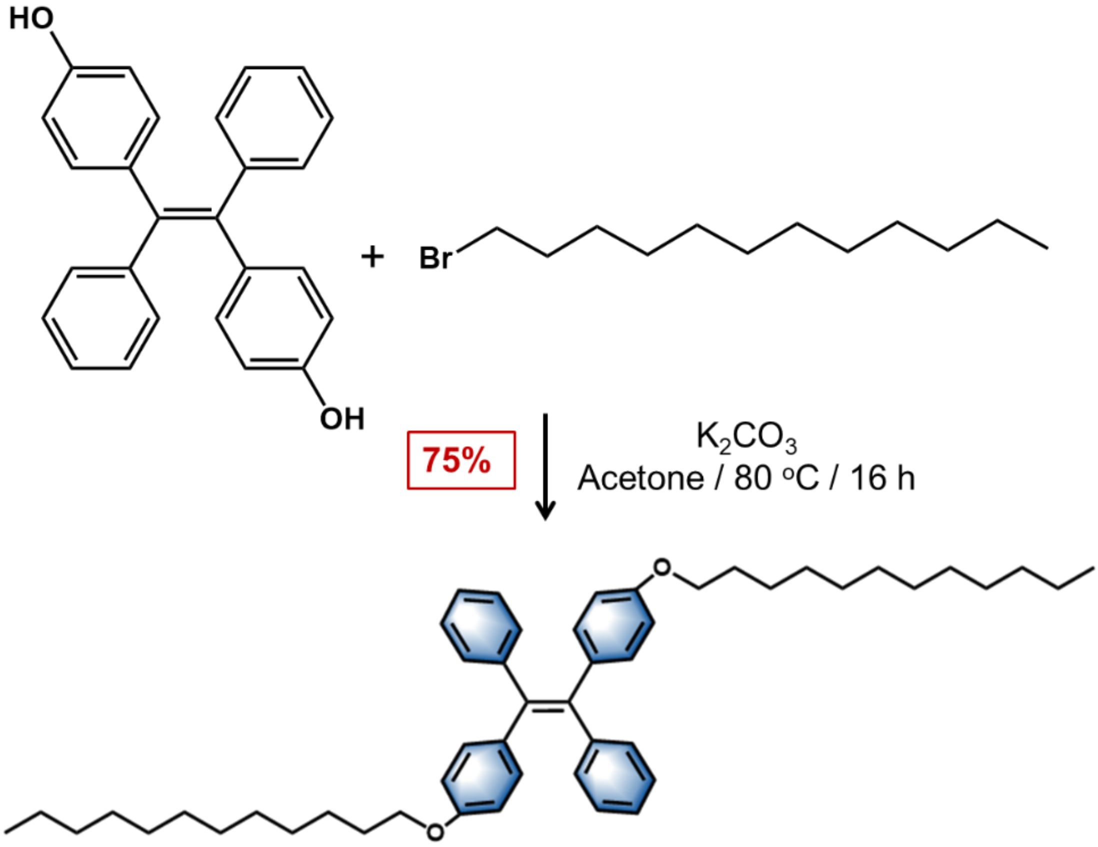

Scheme S1. Synthesis of **TPE-C12**.

Section 2. Figures S1-S14

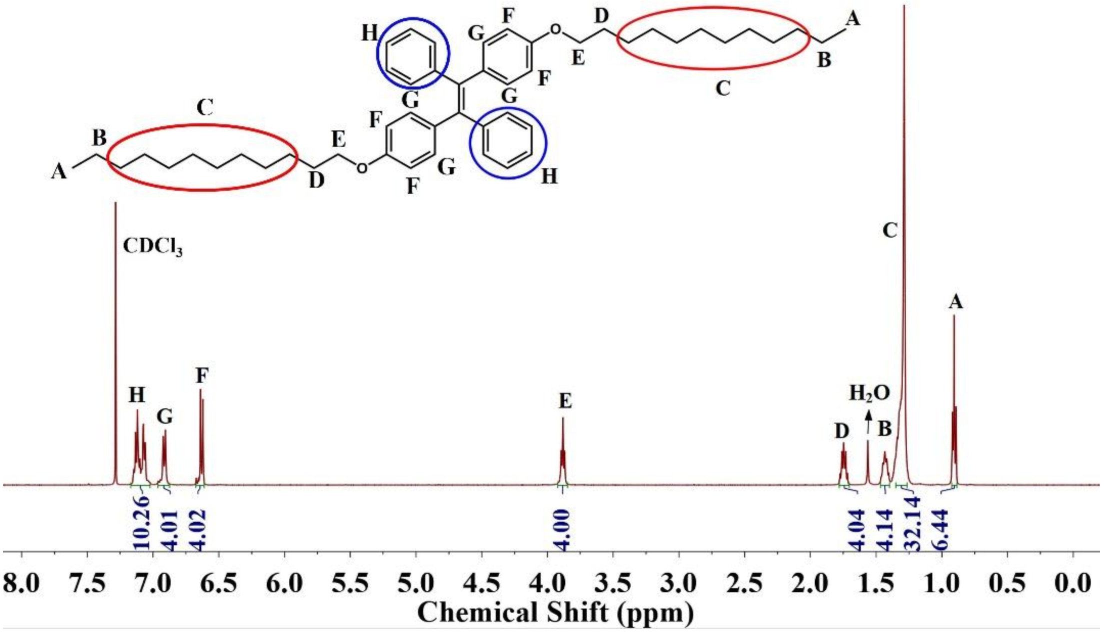

Figure S1. ^{1}H NMR Spectrum (500 MHz, CDCl$_3$, 298 K) of **TPE-C12**.

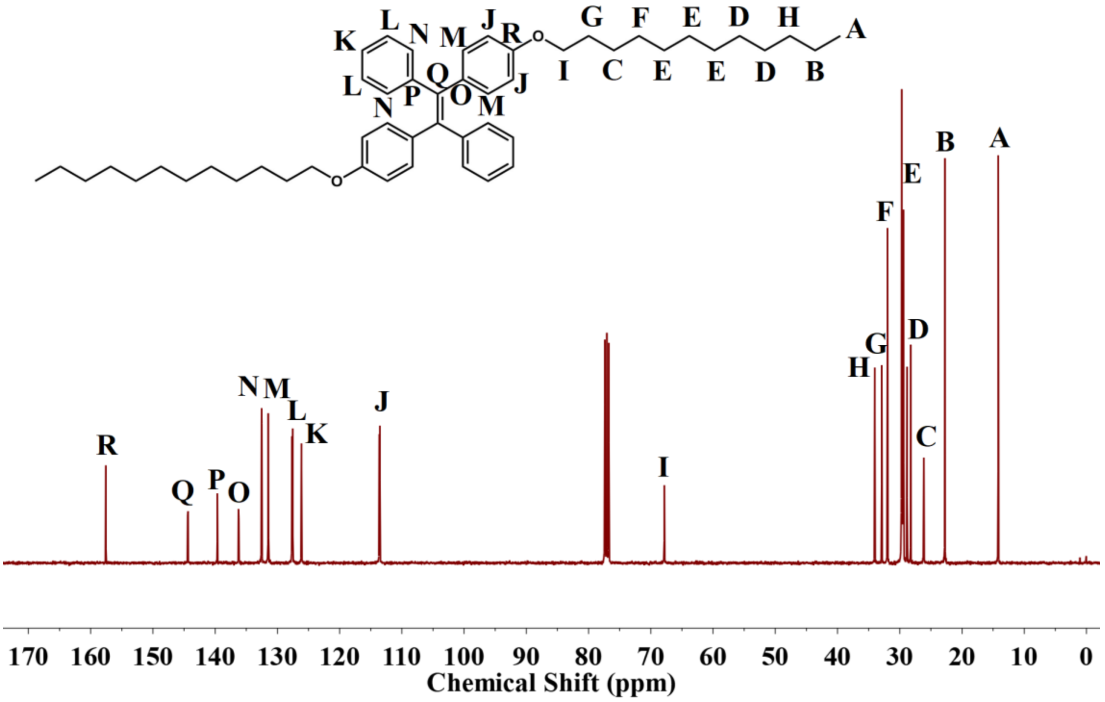

Figure S2. ^{13}C NMR Spectrum (500 MHz, CDCl$_3$, 298 K) of **TPE-C12**.

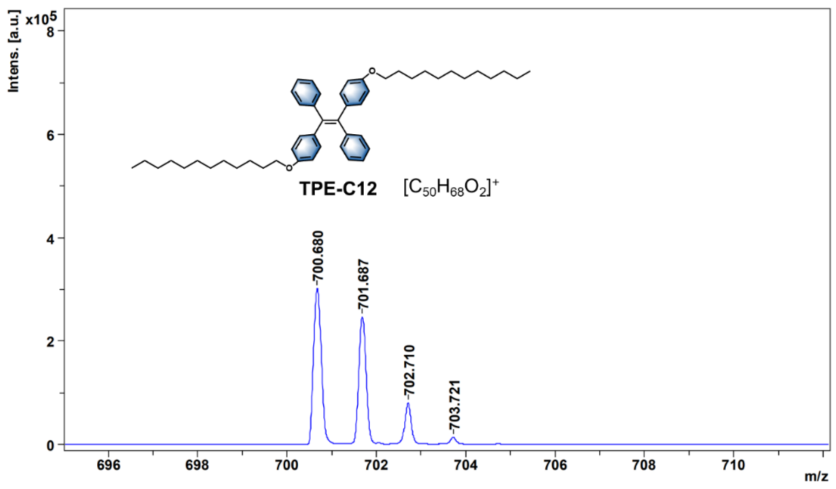

Figure S3. Mass spectrum of **TPE-C12**.

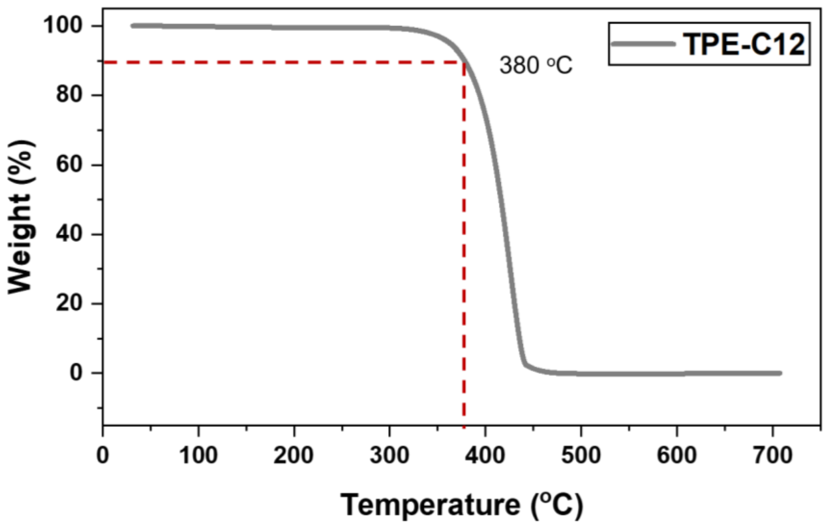

Figure S4. TGA measurement of **TPE-C12** in an air atmosphere with a scan rate of 10 °C/min, with a thermo-decomposing temperature at around 380 °C.

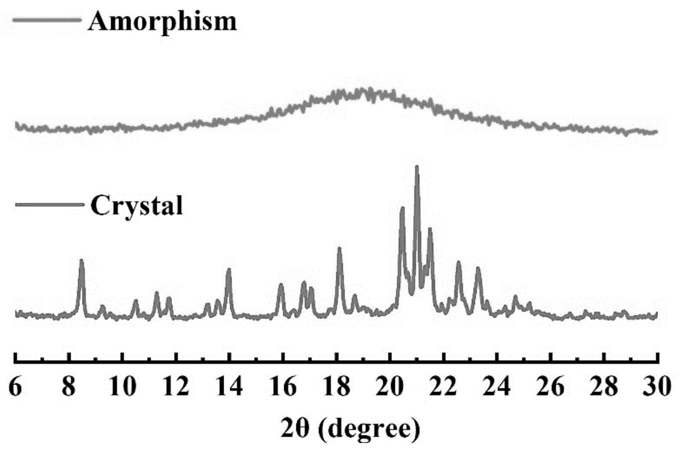

Figure S5. Powder X-ray diffractions of amorphous (heating) and crystalline **TPE-C12**.

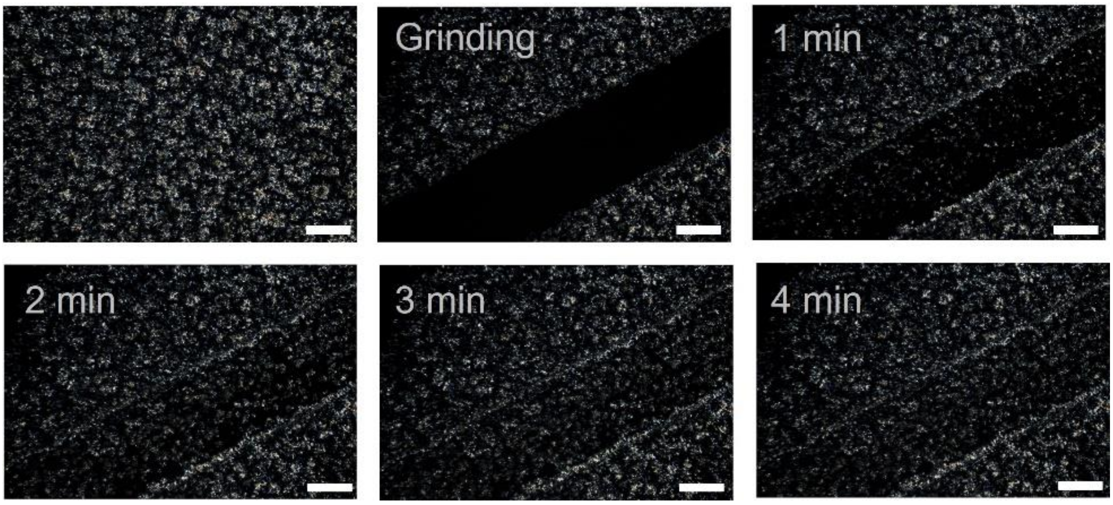

Figure S6. POM images of **TPE-C12** before and after grinding. The scale bars are 20 μm.

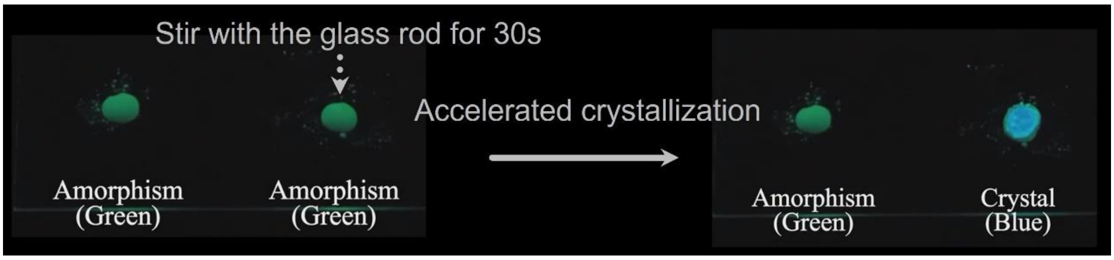

Figure S7. Accelerated crystallization and blue-shift emission of **TPE-C12** after stirring with a glass rod.

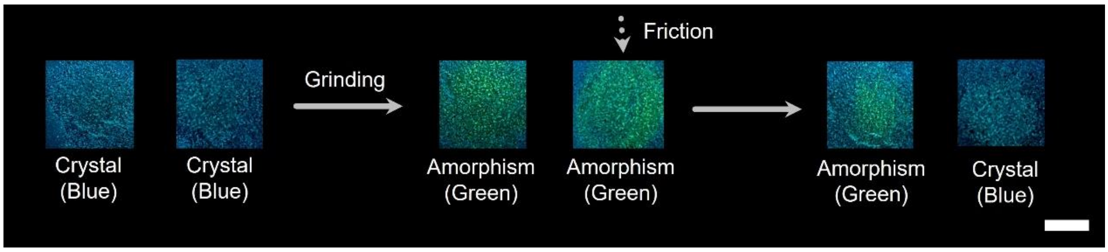

Figure S8. Images of grinding-induced green-shifted emission and friction-induced blue-shifted emission of **TPE-C12**. The scale bar is 0.5 cm.

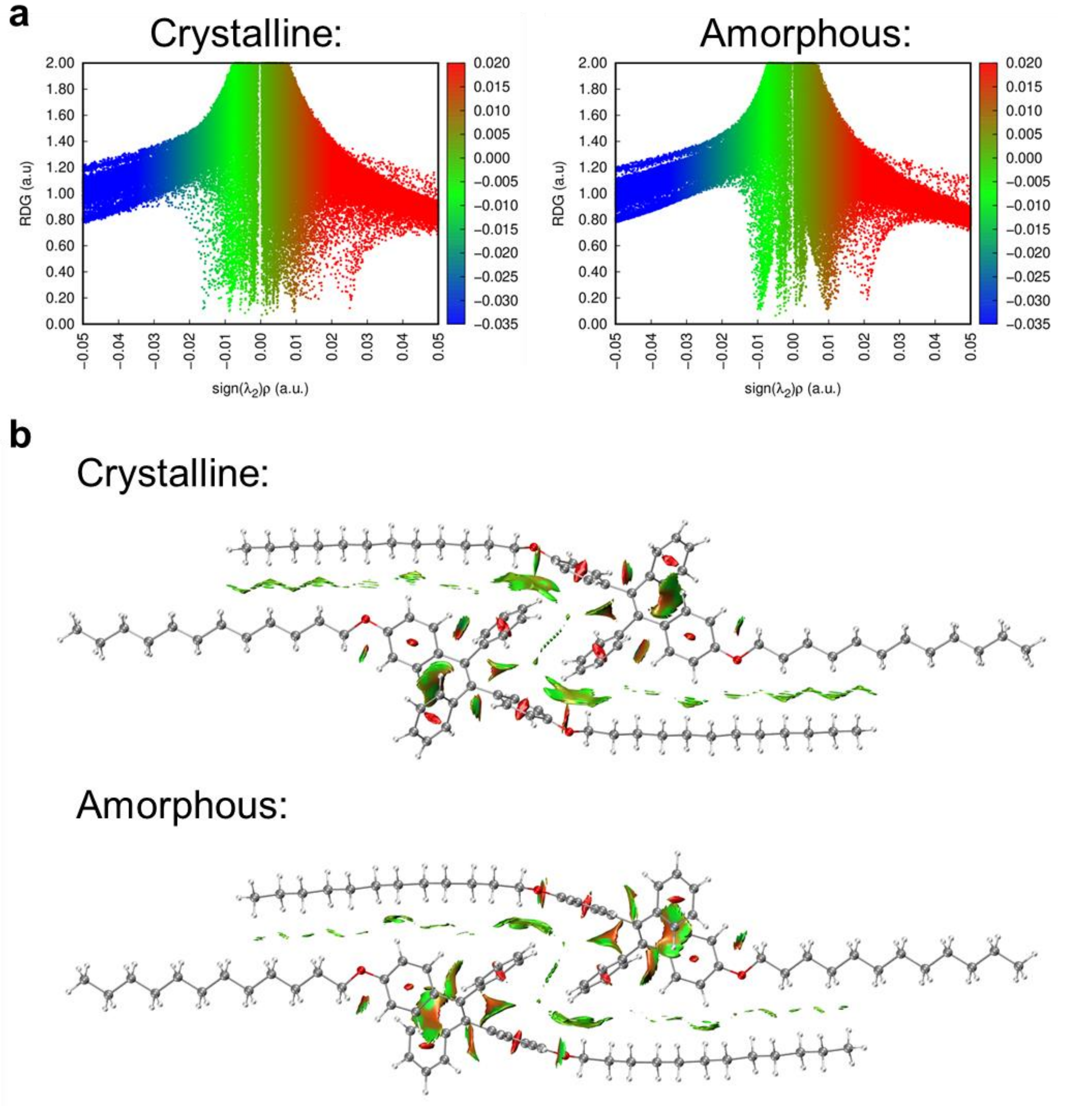

Figure S9. (**a**) The reduced density gradient (RDG) distributions and corresponding (**b**) non-covalent interaction (NCI) analyses of crystalline and amorphous dimers of **TPE-C12**. Crystalline dimers present stronger intermolecular interactions than amorphous one. Green patterns represent van del Waals interactions, while red patterns represent repulsive interactions.

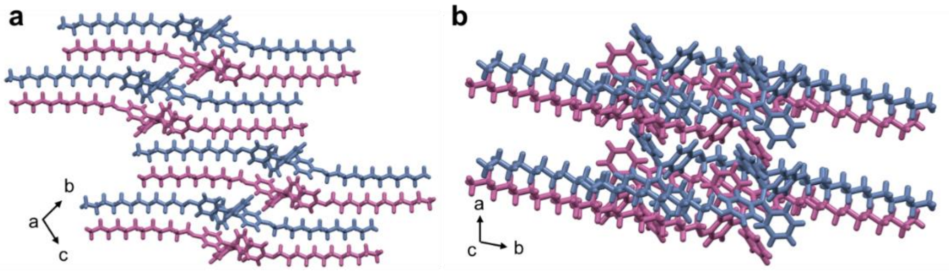

Figure S10. Crystal structure of **TPE-C12** in at a packing view, viewed along (a) *a* and (b) *c* axes.

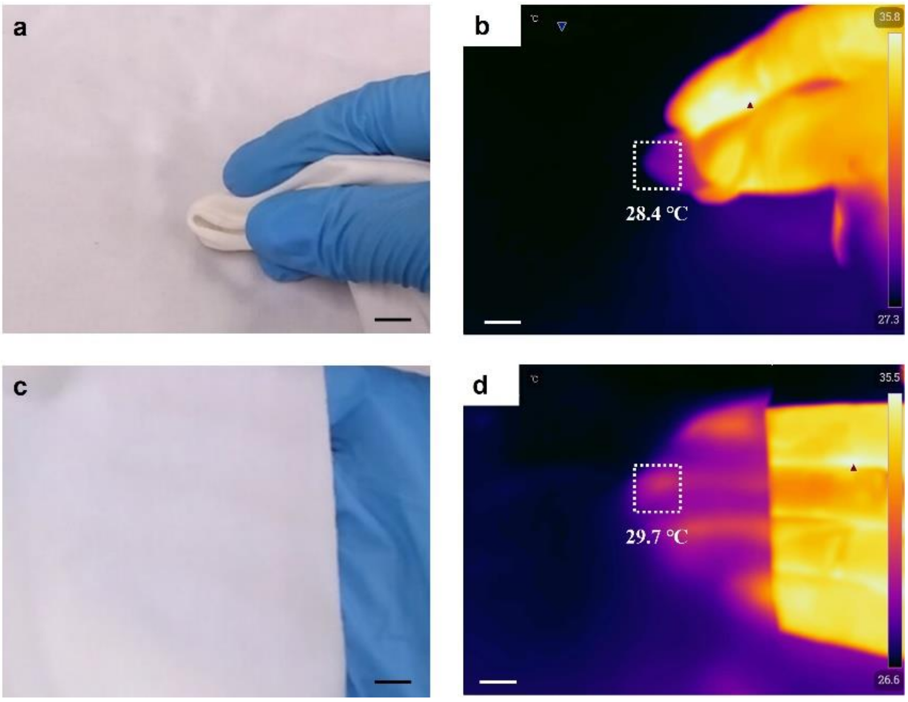

Figure S11. Photographs and infrared thermal images of a fabric holding with the hands. The scale bars are 0.1 cm.

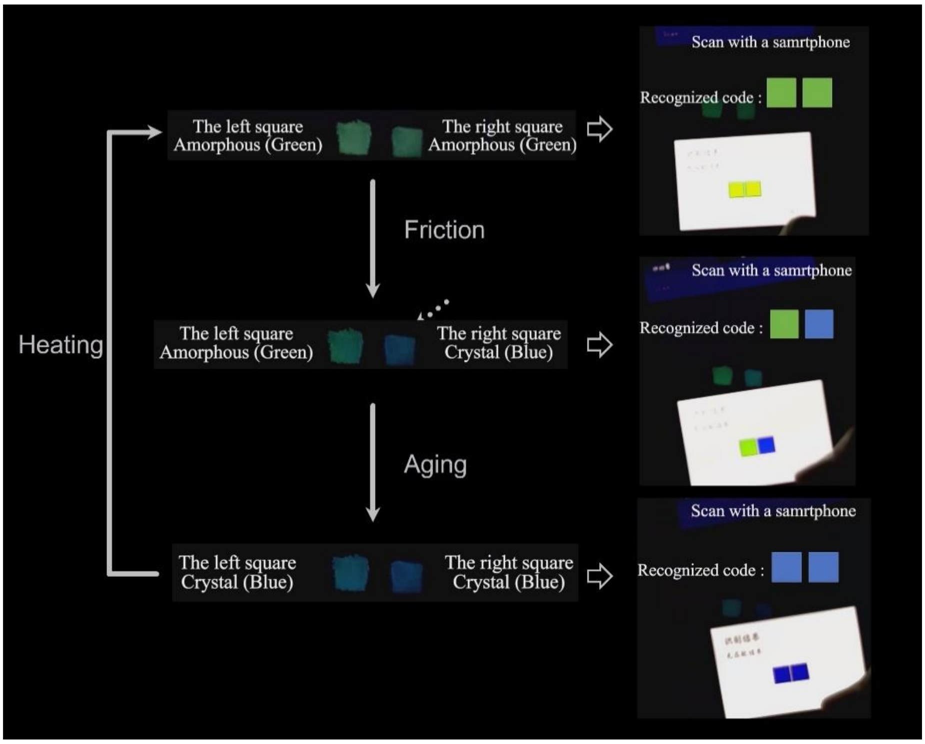

Figure S12. A multilevel information encryption system of a customized application. The square size of scanning code of 1-by-2 squares is 1 cm ×1 cm.

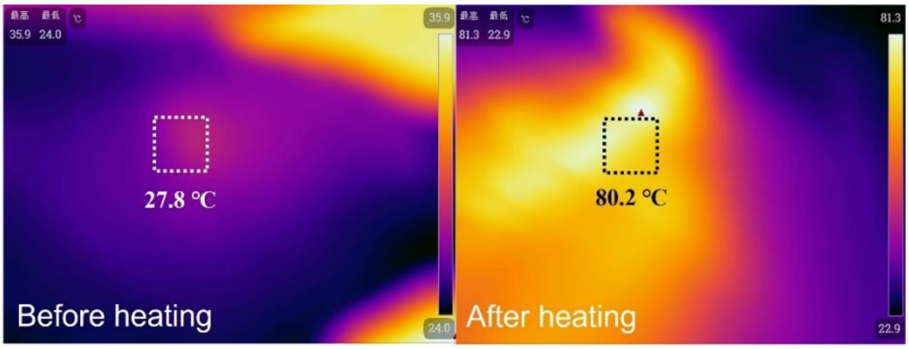

Figure S13. Infrared thermal images captured before and after heating.

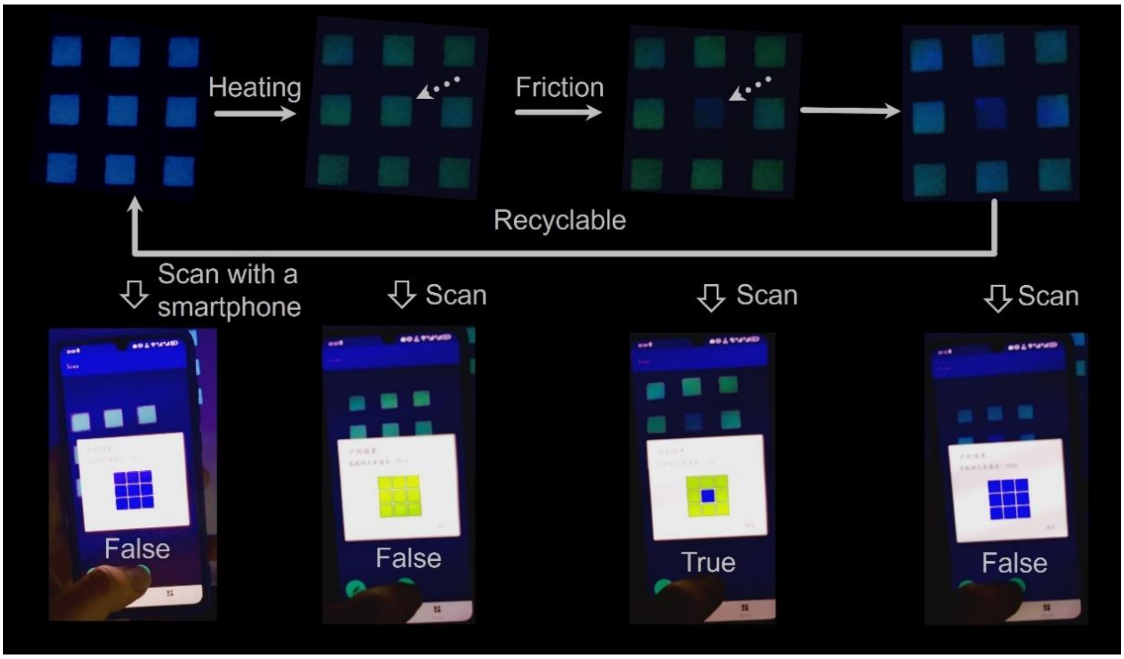

Figure S14. A multilevel information encryption system by a scanning code of 3-by-3 squares. Size of the squares: 1 cm ×1 cm.

Section 3. Table S1

Table S1. Crystal data and structure refinement for **TPE-C12**

Compounds	**TPE-C12**
CCDC	2371736
Empirical formula	$C_{50}H_{68}O_2$
Temperature (K)	170.00
Formula weight	701.04
Crystal system	Triclinic
Space group	$P\bar{1}$
a (Å)	9.759 (4)
b (Å)	10.810 (7)
c (Å)	21.223 (11)
α (°)	102.72 (3)
β (°)	96.845 (16)
γ (°)	100.23 (2)
Volume ($Å^3$)	2120 (2)
Z	2
ρ_{calc} ($g\ cm^{-3}$)	1.098
Absorption coefficient (mm^{-1})	0.064
F (000)	768.0
F′ (000)	768.28
Radiation (Å)	1.54178
R (reflections)	0.0562 (5491)
wR_2 (reflections)	0.1677 (8592)
