# Supplementary material for: Designing High‐Sensitivity Mechanochromic Luminescent Materials Through Friction‐Induced Crystallization Strategy
Source: Adv Sci (Weinh). 2024 Oct 21;11(46):2409974. doi: 10.1002/advs.202409974 (PMC11633541; doi:10.1002/advs.202409974)

checkCIF/PLATON report

Structure factors have been supplied for datablock(s) mo_210825_tpe_2c12_7_0m

THIS REPORT IS FOR GUIDANCE ONLY. IF USED AS PART OF A REVIEW PROCEDURE FOR PUBLICATION, IT SHOULD NOT REPLACE THE EXPERTISE OF AN EXPERIENCED CRYSTALLOGRAPHIC REFEREE.

No syntax errors found. CIF dictionary Interpreting this report

Datablock: mo_210825_tpe_2c12_7_0m

```
Bond precision:   C-C = 0.0030 A          Wavelength=0.71073

Cell:           a=9.759(4)        b=10.810(7)        c=21.223(11)
                alpha=102.72(3)   beta=96.845(16)    gamma=100.23(2)
Temperature:    170 K
```

	Calculated	Reported
Volume	2120(2)	2120(2)
Space group	P -1	P -1
Hall group	-P 1	-P 1
Moiety formula	C50 H68 O2	C50 H68 O2
Sum formula	C50 H68 O2	C50 H68 O2
Mr	701.04	701.04
Dx,g cm-3	1.098	1.098
Z	2	2
Mu (mm-1)	0.064	0.064
F000	768.0	768.0
F000’	768.28	
h,k,lmax	12,13,26	12,13,26
Nref	8676	8592
Tmin,Tmax	0.996,0.997	0.600,0.746
Tmin’	0.994	

```
Correction method= # Reported T Limits: Tmin=0.600 Tmax=0.746
AbsCorr = MULTI-SCAN

Data completeness= 0.990          Theta(max)= 26.367

R(reflections)= 0.0562( 5491)              wR2(reflections)=
                                           0.1677( 8592)

S = 1.024                  Npar= 471
```

The following ALERTS were generated. Each ALERT has the format
test-name_ALERT_alert-type_alert-level.
Click on the hyperlinks for more details of the test.

Alert level C

PLAT906_ALERT_3_C Large K Value in the Analysis of Variance ...... 2.764 Check
PLAT911_ALERT_3_C Missing FCF Refl Between Thmin & STh/L= 0.600 35 Report
PLAT913_ALERT_3_C Missing # of Very Strong Reflections in FCF .... 5 Note

Alert level G

PLAT910_ALERT_3_G Missing # of FCF Reflection(s) Below Theta(Min). 4 Note
PLAT912_ALERT_4_G Missing # of FCF Reflections Above STh/L= 0.600 45 Note
PLAT933_ALERT_2_G Number of OMIT Records in Embedded .res File ... 2 Note
PLAT941_ALERT_3_G Average HKL Measurement Multiplicity .... 3.4 Low
PLAT978_ALERT_2_G Number C-C Bonds with Positive Residual Density. 7 Info

0 **ALERT level A** = Most likely a serious problem - resolve or explain
0 **ALERT level B** = A potentially serious problem, consider carefully
3 **ALERT level C** = Check. Ensure it is not caused by an omission or oversight
5 **ALERT level G** = General information/check it is not something unexpected

0 ALERT type 1 CIF construction/syntax error, inconsistent or missing data
2 ALERT type 2 Indicator that the structure model may be wrong or deficient
5 ALERT type 3 Indicator that the structure quality may be low
1 ALERT type 4 Improvement, methodology, query or suggestion
0 ALERT type 5 Informative message, check

It is advisable to attempt to resolve as many as possible of the alerts in all categories. Often the minor alerts point to easily fixed oversights, errors and omissions in your CIF or refinement strategy, so attention to these fine details can be worthwhile. In order to resolve some of the more serious problems it may be necessary to carry out additional measurements or structure refinements. However, the purpose of your study may justify the reported deviations and the more serious of these should normally be commented upon in the discussion or experimental section of a paper or in the "special_details" fields of the CIF. checkCIF was carefully designed to identify outliers and unusual parameters, but every test has its limitations and alerts that are not important in a particular case may appear. Conversely, the absence of alerts does not guarantee there are no aspects of the results needing attention. It is up to the individual to critically assess their own results and, if necessary, seek expert advice.

Publication of your CIF in IUCr journals

A basic structural check has been run on your CIF. These basic checks will be run on all CIFs submitted for publication in IUCr journals (*Acta Crystallographica*, *Journal of Applied Crystallography*, *Journal of Synchrotron Radiation*); however, if you intend to submit to *Acta Crystallographica Section C* or *E* or *IUCrData*, you should make sure that full publication checks are run on the final version of your CIF prior to submission.

Publication of your CIF in other journals

Please refer to the *Notes for Authors* of the relevant journal for any special instructions relating to CIF submission.

PLATON version of 13/07/2021; check.def file version of 13/07/2021

Datablock mo_210825_tpe_2c12_7_0m - ellipsoid plot

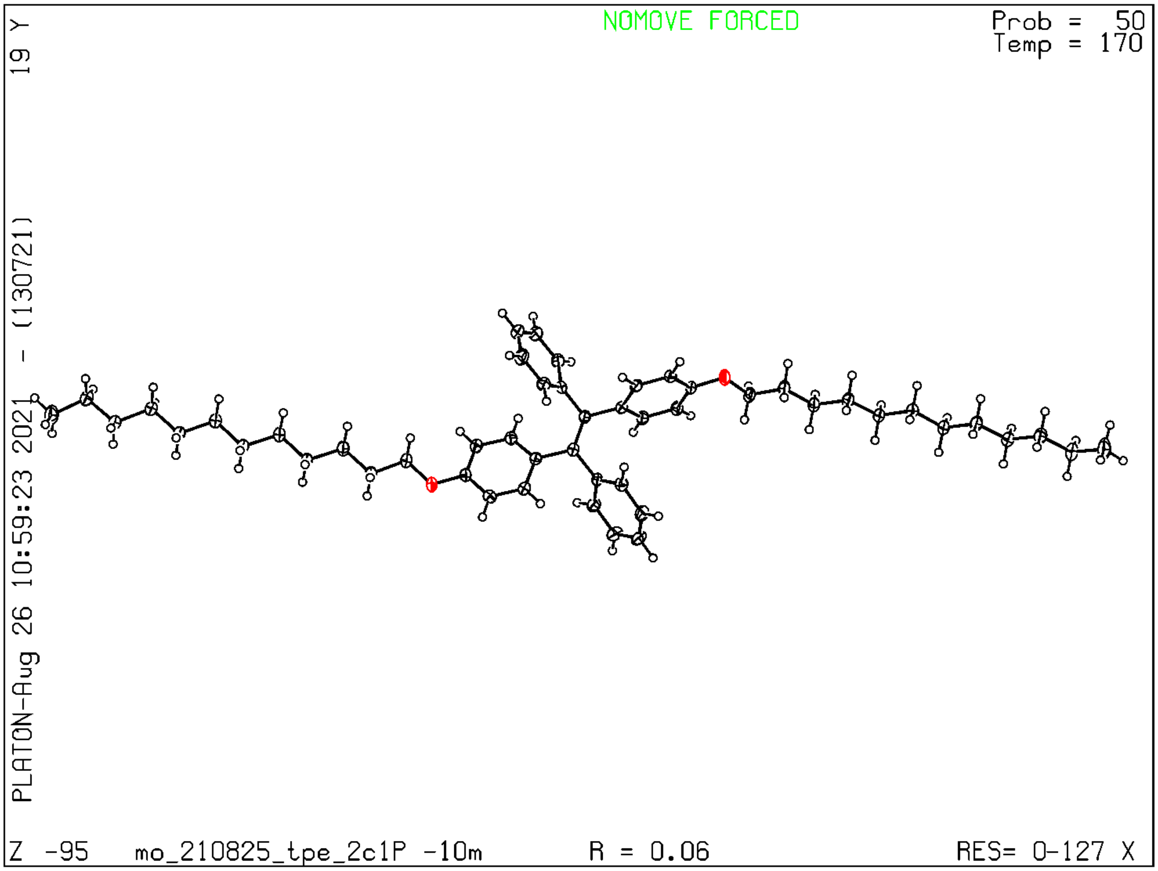

Supplement: Supplementary file 8 — Supporting Information [file ADVS-11-2409974-s006.zip › advs202409974-sup-0008-cif/Checkcif of TPE-C12.pdf]
